# Molecular Basis of Cardioprotective Effect of Antioxidant Vitamins in Myocardial Infarction

**DOI:** 10.1155/2013/437613

**Published:** 2013-07-14

**Authors:** Ramón Rodrigo, Matías Libuy, Felipe Feliú, Daniel Hasson

**Affiliations:** Molecular and Clinical Pharmacology Program, Institute of Biomedical Sciences, Faculty of Medicine, University of Chile, Independencia 1027, Casilla 70058, Santiago 7, Chile

## Abstract

Acute myocardial infarction (AMI) is the leading cause of mortality worldwide. Major advances in the treatment of acute coronary syndromes and myocardial infarction, using cardiologic interventions, such as thrombolysis or percutaneous coronary angioplasty (PCA) have improved the clinical outcome of patients. Nevertheless, as a consequence of these procedures, the ischemic zone is reperfused, giving rise to a lethal reperfusion event accompanied by increased production of reactive oxygen species (oxidative stress). These reactive species attack biomolecules such as lipids, DNA, and proteins enhancing the previously established tissue damage, as well as triggering cell death pathways. Studies on animal models of AMI suggest that lethal reperfusion accounts for up to 50% of the final size of a myocardial infarct, a part of the damage likely to be prevented. Although a number of strategies have been aimed at to ameliorate lethal reperfusion injury, up to date the beneficial effects in clinical settings have been disappointing. The use of antioxidant vitamins could be a suitable strategy with this purpose. In this review, we propose a systematic approach to the molecular basis of the cardioprotective effect of antioxidant vitamins in myocardial ischemia-reperfusion injury that could offer a novel therapeutic opportunity against this oxidative tissue damage.

## 1. Introduction

Acute myocardial infarction (AMI) is the leading cause of mortality worldwide. In 2008 ischemic heart disease accounted for 7.25 million deaths worldwide (12.8%), according to the WHO. It is of relevance to consider not only its impact in mortality, but also the impairment in the life quality of patients surviving this vascular accident. During the last decades, therapies in use have shown a significant mortality reduction in myocardial infarction patients [1–4]. However, such beneficial effects are still of limited efficacy, and new therapies are currently being investigated. Systemic thrombolysis and percutaneous coronary angioplasty (PCA) have been used to recover the myocardial perfusion, with the latter being the most successful, as it allows to reestablish the blood flow in the cardiac zones affected by the occlusion of a branch of the coronary artery. Nevertheless, as a consequence of this procedure, the ischemic zone is reperfused, giving rise to an ischemia-reperfusion event that generates increased production of reactive oxygen species (oxidative stress) [5], thus enhancing the previously established tissue damage (lethal reperfusion), as these reactive species attack biomolecules such as lipids, DNA, and proteins and trigger cell death pathways [6]. Studies on animal models of AMI suggest that lethal reperfusion accounts for up to 50% of the final size of a myocardial infarct, a part of the damage likely to be prevented [7]. Although a number of strategies have been aimed at to ameliorate lethal reperfusion injury, up to date the beneficial effects in clinical settings have been disappointing. The use of antioxidant vitamins could be a suitable strategy with this purpose, but oral administration does not allow reaching the plasma levels required to counteract the effects of oxidative stress [8]. Alternatively, short episodes of ischemia before total reperfusion such as short balloon inflations before final reperfusion during coronary angioplasty may have protective effects [9]. In a rabbit model, the administration of ascorbate aggravates damage, likely through the abrogation of the endogenous enzymatic antioxidant response triggered by short episodes of ischemia [10]. Some protocols using intravenous antioxidant vitamins have failed to achieve a significant amelioration of infarct size. Reinforcement of the antioxidant defense system should be expected to protect the myocardium against the reperfusion injury. Indeed, at present, no study with this purpose has used ascorbate in doses high enough to scavenge superoxide anion. Interestingly, even high doses reaching plasma vitamin C levels higher than 10 mmoles/L have been administered in other clinical settings [11]. This dose and higher ones proved to be remarkably safe in a recent meta-analysis [12]. In this review we propose a systematic approach to the molecular basis of antioxidant vitamin treatment against ischemia-reperfusion injury in myocardial infarction.

## 2. Role of Ischemia-Reperfusion in Myocardial Infarction 

### 2.1. Oxidative Stress in Myocardial Ischemia-Reperfusion 

#### 2.1.1. Major Role of Oxidative Stress

Oxidative stress constitutes a unifying mechanism of injury of many types of disease processes; it occurs when there is an imbalance between the generation of reactive oxygen species (ROS) and the antioxidant defense systems in the body so that the latter become overwhelmed [13]. ROS are a family of highly reactive species that are formed either enzymatically or nonenzymatically in mammalian cells and causing cell damage either directly or through behaving as intermediates in diverse cell signaling pathways. Antioxidant defenses can be divided into enzymatic and nonenzymatic. Enzymatic antioxidant defenses mainly include superoxide dismutase (SOD), glutathione peroxidase (GSH-Px), catalase (CAT), and thioredoxin peroxidase, among others. Non-enzymatic antioxidant defenses include a variety of biological molecules, such as ascorbic acid (vitamin C), *α*-tocopherol (vitamin E), reduced glutathione (GSH), coenzyme Q10, cysteine, carotenoids, flavonoids, polyphenols, and other various exogenous antioxidants [14, 15]. Polyphenols act as antioxidants both through the prevention of damage from ROS and their iron chelating ability [16], further enhancing the *in vivo* antioxidant potential. The first line of cellular defense against oxidative injury in the heart as well as most tissues includes the antioxidant enzymes CAT, SOD, and GSH-Px [15]. There are many mechanisms through which antioxidants may act such as (1) scavenging reactive oxygen species or their precursors, (2) inhibiting the formation of ROS, (3) attenuating the catalysis of ROS generation via binding to metal ions, (4) enhancing endogenous antioxidant generation, and (5) reducing apoptotic cell death by upregulating the antideath gene Bcl-2 [14].

In normal physiological conditions, oxygen-free radical production is usually kept under homeostatic control by endogenous free radical scavengers known as antioxidants. However, during ischemia there is a loss of antioxidant enzyme function, together with leakage of antioxidant enzymes into the extracellular fluid. The levels of myeloperoxidase (MPO), a strong prooxidant enzyme, have a prognostic role in acute coronary syndromes. MPO levels were associated with no-reflow phenomenon or impaired myocardial microcirculation in STEMI patients. Neutrophil activation occurring in ischemia-reperfusion is one of the major determinants of vascular impairment in myocardial tissue, and MPO is just a marker of neutrophil activation [28]. On reperfusion, the enzymes are then washed out, further depleting the available control over free radical production, and as a result, the unbalanced burst of free radicals on reperfusion (respiratory burst) easily overwhelms the available counteractive enzymes so the control of ROS generation is lost [14].

In addition, AMI is a clinical model of oxidative stress by ischemia-reperfusion. Reactive oxygen species (ROS) are major initiators of myocardial damage during ischemia/reperfusion. Accordingly, AMI is usually initiated by myocardial ischemia due to coronary artery obstruction. In pathophysiological conditions, sources of ROS include the mitochondrial respiratory electron transport chain, xanthine oxidase activation due to ischemia-reperfusion, the respiratory burst associated with neutrophil activation, and arachidonic acid metabolism. Several studies have proposed the essential role of ROS in the pathogenesis of myocardial ischemia-reperfusion injury. ROS including hydrogen peroxide (H_2_O_2_), superoxide radical, hydroxyl radical (OH*·*), and peroxynitrite (ONOO^−^) have been shown to increase upon reperfusion of the heart following ischemia [29]. An increase in the formation of ROS during ischemia-reperfusion was also reported by using the electron paramagnetic resonance technique. ROS seem to increase significantly after a few minutes of reperfusion, but its increase during ischemia alone is still controversial. On the basis of these changes it has been suggested that the increase of H_2_O_2_ production and other ROS during ischemia-reperfusion leads to lipid peroxidation and sulfhydryl group oxidation [30].

#### 2.1.2. Pathophysiology of Myocardial Ischemic Injury

A series of biochemical and metabolic changes in myocardial tissue occur due to deprivation of oxygen and nutrient supply during ischemia. Consequently mitochondrial damage and ATP depletion impair myocardial contractile function [9]. Anaerobic glycolysis due to the absence of oxygen results in the accumulation of lactate and intracellular pH reduction (to <7.0). The latter activates the Na^+^-H^+^ ion exchanger, thus extruding protons from the cell in exchange for Na^+^ entry. Furthermore the impaired function of (Na + K)-ATPase contributes to exacerbate the intracellular Na^+^ and Ca^2+^ overload [31].

#### 2.1.3. Pathophysiology of Myocardial Reperfusion Injury

**Figure 1:**
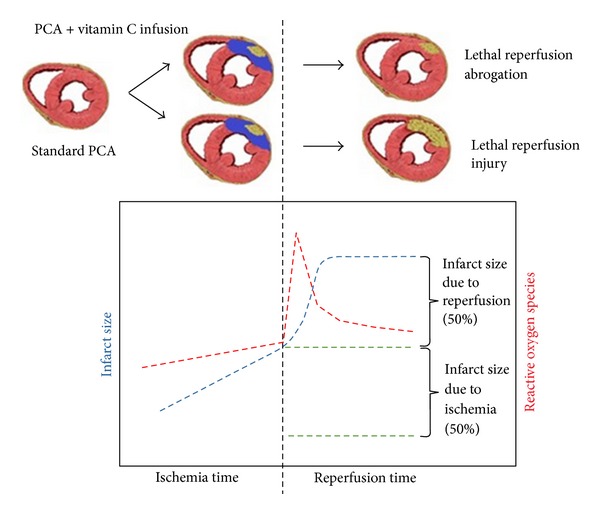
Schema representing the time course of the effects of ischemia reperfusion. Upon reperfusion there is an oxidative burst, which corresponds to a marked increase in infarct size. Counteracting this process could account for a decrease of up to 50% infarct size.

The level of tissue oxygenation increases following restoration of blood flow, which is followed by a burst of ROS generation that leads to the syndrome of reperfusion injury [5]. Neutrophils are the primary source of ROS during reperfusion, although endothelial cells and cardiomyocytes can also generate this reactive species. Increased ROS production is mainly due to activation of xanthine oxidase in endothelial cells, mitochondrial electron transport chain reactions in cardiomyocytes, and NADPH oxidase in inflammatory cells [32]. Under these conditions, the enzymatic antioxidant effect is relevant against the detrimental effects of ROS. Therefore, it should be expected that a reinforcement of the antioxidant defense system through ROS scavengers results in a cardioprotective effect during the myocardial reperfusion (Figure 1). After an ischemic episode of the myocardium, left ventricle remodeling is known to occur; although its underlying mechanism is multifactorial, ROS and inflammatory cytokines may cause cardiodepressive reaction [33–35]. It is of interest to remark that ROS also stimulate the production of inflammatory cytokines and, in turn, inflammatory cytokines stimulate ROS formation. In chronic stage, ROS and inflammatory cytokines activate the matrix metalloproteinases [36, 37], thereby eliciting degradation of collagens which may cause a slippage in myofibrillar alignment causing left ventricular dilatation [38].

The ischemia-reperfusion injury includes a series of events: (a) reperfusion arrhythmias, (b) microvascular damage, (c) myocardial stunning “reversible mechanical dysfunction,” and (d) cell death, all of which may occur either together or separately [39]. There are two main hypotheses, namely, oxidative stress and Ca^2+^-overload, which have been proposed to explain the pathogenesis of ischemia-reperfusion injury [40, 41]. Both these mechanisms are most likely related to each other but it is not known whether they operate simultaneously or if one precedes the other. With respect to this, oxidative stress, which is usually associated with increased formation of ROS, modifies phospholipids and proteins leading to lipid peroxidation and thiol groups oxidation; these changes are considered to alter membrane permeability and configuration in addition to producing functional modification of various cellular proteins [42]. Oxidative stress may result in cellular defects including a depression in the sarcolemmal Ca^2+^-pump ATPase and (Na + K)-ATPase activities, changes leading to decreased Ca^2+^-efflux and increased Ca^2+^-influx, respectively [43]. Oxidative stress has also been reported to depress the sarcoplasmic reticulum Ca^2+^-pump ATPase and thus inhibit Ca^2+^ sequestration from the cytoplasm in cardiomyocytes. These alterations were markedly reduced by antioxidants such as catalase and superoxide dismutase [44]. The depression in Ca^2+^-regulatory mechanism by ROS ultimately results in intracellular Ca^2+^ ([Ca^2+^]_i_) overload and cell death. On the other hand, an increase in [Ca^2+^]_i_ during ischemia induces the conversion of xanthine dehydrogenase to xanthine oxidase and subsequently results in generating superoxide radicals [44].

### 2.2. Other Mediators of Myocardial Injury

#### 2.2.1. Inflammation in Ischemia-Reperfusion

ROS generation could occur through several enzymatic reactions in cell types such as endothelial, inflammatory, and cardiomyocyte cells. Among these enzymatic sources, much attention has been placed on xanthine oxidase in endothelial cells, NADPH oxidase in inflammatory cells and the mitochondrial electron transport chain reaction in cardiomyocytes using either *in vivo* models of ischemia-reperfusion or cultured endothelial cells and cardiomyocytes after hypoxia-reoxygenation [32, 45, 46]. It has been proposed that a burst of ROS from endothelial cells, and cardiomyocytes during early reperfusion can influence nearby neutrophils, setting up a local cycle of amplified cellular response through released inflammatory mediators. Furthermore, neutrophils become sensitized (primed) to activating factors, such as chemotactic cytokines, after they adhere to the endothelium, and thus generate much greater quantities of ROS. Specifically, the chemokine interleukin-8 appears to have a fundamental role in regulating neutrophil localisation in ischaemic myocardium. In mice, CXCL2, the homologue of human interleukin-8, is upregulated in reperfused myocardium [47]. The chemokine response in ischaemic tissues may be induced by various factors, including ROS, cytokines (e.g., tumour necrosis factor (TNF)-*α*), the complement system, and nuclear factor *κ*-light-chain-enhancer of activated B cells (NF-*κ*B) activation, a major proinflammatory transcription factor [48]. After the initial burst of ROS at the onset of reperfusion, later events such as transendothelial migration of neutrophils and macrophages might participate in delayed ROS generation during reperfusion [49, 50]. Activated neutrophils produce superoxide as a cytotoxic agent as part of the respiratory burst via the action of membrane-bound NADPH oxidase on molecular oxygen. Despite the fact that superoxide anion per se is not a potent oxidant, its interaction with nitric oxide (NO) can lead to the powerful oxidant ONOO^−^. In addition it should be mentioned that transition metal ions, such as iron, could give rise to the very harmful Fenton and Haber-Weiss reactions. Even small amounts of intracellular nonbound iron (labile iron pool) may interact with superoxide leading to the formation of extremely reactive hydroxyl radical. Also, in the presence of iron the antioxidant vitamins may act as prooxidants [51]. Neutrophils also produce the free radical NO that can react with superoxide to produce ONOO^−^, a powerful oxidant, which may decompose to form OH*·*. Prospective epidemiological studies have shown that serum levels of C-reactive protein (CRP), a biomarker of inflammation, are a strong predictor of cardiovascular ischaemia-reperfusion injury cycle events, such as myocardial infarction, postoperative atrial fibrillation and stroke [52]. Several studies revealed an independent association of high plasma CRP levels with adverse prognosis in acute myocardial infarction patients. Interestingly, preconditioning was found to inhibit postischaemic CRP increases in a rat model of acute myocardial infarction [53].

#### 2.2.2. Effect of pH on Cardiomyocyte Function

A decreased intracellular pH during ischemia is rapidly restored to physiological pH by the washout of lactate during reperfusion. It is of interest to note that pH shift contributes to the cardiomyocyte death of lethal myocardial reperfusion injury [54] by permitting mitochondrial permeability transition pore (mPTP) opening and cardiomyocyte rigor hypercontracture in the first few minutes of reperfusion. Also, it has been described that acidosis protects against lethal anoxic injury and that a rapid return from acidotic to physiologic pH significantly contributes to reperfusion injury to cardiac myocytes, a “pH paradox” [55].

#### 2.2.3. Intracellular Ca^2+^ Overload 

Dysregulation of Ca^2+^ homeostasis has long been implicated to play an important role in cell injury. Pathological Ca^2+^ overload and calcification are frequently features of tissue ischemia and infarction, and increased Ca^2+^ activates a number of phosphatases, proteases, and nucleases [56]. The effects of calcium overload in acute myocardial ischemia are due to disruption of the plasma membrane, oxidative stress-induced damage to the sarcoplasmic reticulum, and mitochondrial reenergization. Mitochondrial re-energization allows the recovery of the mitochondrial membrane potential that drives the entry of Ca^2+^ into mitochondria via the mitochondrial Ca^2+^ uniporter and subsequently induces the opening of the mPTP [57]. Ca^2+^ release from the endoplasmic reticulum may flood the cytosol with free Ca^2+^, possibly leading to activation of degradative processes and dysfunction of other organelles, particularly mitochondria [58]. In addition, calcium overload besides other detrimental effects increases the arrhythmic risk by provoking afterdepolarizations in cardiac cells. Recent evidence suggests that blockade of calcium current was highly effective in suppression of early afterdepolarizations [59].

#### 2.2.4. The Role of mPTP in Myocardial Ischemia

Many chemicals and radicals are inducers that promote the mPTP opening, thus decreasing the threshold amount of Ca^2+^ needed in that process [56]. Opening of mPTP results in mitochondrial membrane depolarization and uncoupling of oxidative phosphorylation, leading to ATP depletion and cell death [60, 61]. In has been shown that, in settings of acute myocardial ischemia reperfusion injury, the mPTP remains closed during ischemia and only opens at reperfusion in response to mitochondrial Ca^2+^ and phosphate overload, oxidative stress and relative ATP depletion, and rapid pH correction [62]. 

### 2.3. Cell Death Signaling Pathways: Apoptosis, Necrosis, and Autophagy

In AMI, ROS are generated in the ischemic myocardium especially after reperfusion. ROS directly injure the cell membrane and cause cell death [6]. However, ROS also stimulate signal transduction to elaborate inflammatory cytokines, for example, (TNF)-*α*, interleukin (IL)-1*β*, and -6, in the ischemic region and surrounding myocardium as a host reaction. Inflammatory cytokines also regulate cell survival and cell death in the chain reaction with ROS. Apoptosis or programmed cell death is a distinct form of destruction of the cell which is associated with synthesis of enzymes that degrade and fragment its own DNA. Briefly, the signal pathway of apoptosis involves the stimulation of cell membrane death receptors (Fas) which leads to the activation of caspases (aspartate-specific proteases), protein cleavage, DNA fragmentation, and cell death. Several studies have shown that myocardial ischemia-reperfusion is associated with an increase in apoptotic cells [63]. However, the exact mechanisms underlying the induction of this apoptotic process and the long-term consequences of this process in myocardial ischemia-reperfusion are not completely understood. Exposure of cultured rat cardiomyocytes to lower doses of an exogenous ROS-generating system, such as H_2_O_2_ and superoxide anion, caused release of cytochrome c and activation of caspase-3 and triggered apoptotic cell death [64]. Various studies suggest that release of ROS from activated neutrophils [65] and macrophages [66, 67] may contribute to the early and progressively increasing apoptosis. A significant linear relationship between the number of apoptotic myocytes and transmigrated neutrophils, as well as macrophages, was also observed during early and prolonged reperfusion [68, 69]. Updated information suggests that ischemia followed by reperfusion significantly induces myocardial injury by an apoptotic death pathway. To understand potential signaling mechanisms involved in ROS-triggered apoptosis, recent reports have shown that intracellular Ca^2+^ overload and enhanced activity of the mitogen-activated protein kinase (MAPK) family during reperfusion can participate in induction of ROS-mediated apoptosis in addition to necrosis and eventually could be determinant of the infarct size [70]. 

Cell death was once viewed as unregulated. It is now clear that at least a portion of cell death is a regulated cell suicide process. This type of death can exhibit multiple morphologies. One of these, apoptosis, has long been recognized to be actively mediated, and many of its underlying mechanisms have been elucidated. Moreover, necrosis, the traditional example of unregulated cell death, is also regulated in some instances. Autophagy is usually a survival mechanism but can occur in association with increased ROS leading to cell death. Little is known, however, about how autophagic cells die [71]. Apoptosis, necrosis, and autophagy occur in cardiac myocytes during myocardial infarction, ischemia-reperfusion, and heart failure. Pharmacological and genetic inhibition of apoptosis and necrosis lessens infarct size and improves cardiac function in these disorders [72].

#### 2.3.1. Apoptosis and Necrosis 

Apoptosis is a highly controlled cell death process that is autonomously committed by both healthy and sublethally injured cells in response to physiological or pathological stimuli, including ischemia-reperfusion events. Necrotic cell death is a widely recognised property of ischemic cell death and is clinically diagnosed by documenting myocyte release of cytosolic constituents, such as creatine kinase MB, troponins, and other proteins. However, apoptosis has only been implicated in the pathogenesis of several acute and chronic conditions affecting the cardiovascular system in the last decade [73]. The loss of endothelial cells precedes and may predispose cardiomyocytes to undergo apoptosis [74], indicating that salvaging endothelial cells is of paramount importance. Whether myocyte apoptosis is initiated during ischemia but dependent on reperfusion or whether it is a feature of reperfusion injury requires further study. Reperfusion appears to accelerate apoptosis when compared with permanent occlusion [75]. In contrast to the modest, chronically elevated levels of cell death during heart failure, myocardial infarction is characterised by a large burst of cardiac myocyte death that is usually complete within 24 hours. Active caspases cleave vital substrates in the cell, such as actin, actinin, *β*-myosin heavy chain, myosin light chain, tropomyosin, and cardiac troponins, leading to cellular demise [76]. The “intrinsic” pathway utilises mitochondria to induce cell death by opening the mPTP or rupturing the outer mitochondrial membrane, both of which trigger the sudden and complete release of cytochrome c and other proteins from the intermembrane mitochondrial space into other cellular compartments. The “intrinsic” pathway is primarily activated in cardiac myocytes by cellular stimuli, such as hypoxia, ischemia-reperfusion, and oxidative stress, which perturb the mPTP and increase the permeability of the outer and inner mitochondrial membranes [77]. Once released, cytochrome c binds to the cytosolic protein Apaf1 and facilitates formation of the “apoptosome” complex, which results in caspase-9 activation that provokes caspase-3 activation [78]. Smac/DIABLO indirectly activates caspases by sequestering caspase-inhibitory proteins, while the mitochondrial release of endonuclease-G and apoptosis-inducing factor results in their translocation into the nucleus where they directly or indirectly facilitate DNA fragmentation [79]. The “extrinsic” apoptotic pathway involves the death-receptor Fas pathway. Binding of the transmembrane protein Fas to its cognate receptor induces receptor clustering and the formation of a death-inducing signalling complex. Cardiac overexpression of the Fas ligand results in accentuated apoptosis *in vitro*, whereas Lpr mice, which lack Fas, display less apoptosis and reduced infarct size in ischemia-reperfusion studies [80].

Apoptotic cell death can transition to necrosis during oxidative stress by two possible mechanisms. First, the inactivation of caspases due to oxidation of their active site thiol groups by oxidants or S-nitrosylation can lead to necrosis-like cell death in fatally damaged cells [81]. Second, a drop in ATP levels due to the failure of mitochondrial energy production by oxidants can cause apoptosis to change to necrosis [82]. In addition, it was recently found that the proapoptotic protein Bnip3 is associated with mitochondrial dysfunction and cell death. Bnip3 is also a potent inducer of autophagy in many cell types, including adult cardiac myocytes. Bnip3 overexpression induces selective removal of mitochondria in cardiac myocytes and triggers induction of autophagy independent of calcium, ROS generation, and mPTP opening [83]. Furthermore, it was recently reported that angiotensin II induces mitochondrial autophagy and biogenesis through mitochondrial ROS in the mouse heart [84].

#### 2.3.2. Autophagy

In contrast to necrosis and apoptosis, autophagy is primarily a survival mechanism. Cellular oxidative stress and ROS have been reported to serve as important autophagic stimuli during periods of ischemia-reperfusion [85]. Autophagic degradation and removal of damaged oxidised proteins in response to low to moderate oxidative stress are reportedly beneficial for cells. Conversely, severe oxidative stress and increasing amounts of ROS may activate signalling pathways that lead to autophagy-induced cell death. Whether autophagy promotes cell survival or death depends upon the severity and degree of stress in the cellular environment [86]. During the initial period of ischemia, enzyme xanthine oxidase is formed, and substrates for xanthine oxidase (hypoxanthine and xanthine) accumulate. Upon reperfusion, the reintroduction of O_2_ leads to xanthine oxidase-mediated superoxide generation due to the presence of xanthine and hypoxanthine [87]. During this period, ATP generation decreases and results in the phosphorylation of 5′adenosine monophosphate-activated protein kinase (AMPK), which leads to autophagosome formation through inhibition of mammalian target of rapamycin (mTOR) [88]. Meanwhile, ROS damage organelles and cytosolic proteins and cause mitochondrial lipid peroxidation, all of which exacerbate autophagy [89]. Additionally, antioxidant enzymes, such as CAT and SOD, are targeted by autophagosomes. This ultimately leads to the induction of cell death and is thus detrimental to tissue function. Furthermore, AMPK activity decreases during reperfusion, thus increasing autophagic death and upregulating beclin-1 [90]. Autophagy has been reported to be involved in cardioprotection against lethal ischemic injury; thus, repetitive ischemia by coronary stenosis or occlusion enhances autophagy and subsequent cardioprotection when compared with classical ischemia-reperfusion insult [91]. Aside from the key role that the chemokine monocyte chemoattractant protein-1 (MCP-1) plays in cardiac damage following ischemia, it also mediates autophagy through MCP-1-induced protein (MCPIP), a novel zinc-finger protein that has transcription factor-like activity [92]. MCPIP stimulates inducible NO synthase, translocation of the NADPH oxidase subunit phox47 from the cytoplasm to the membrane, ROS production, induction of endoplasmic reticulum stress markers HSP40, and autophagy, as indicated by beclin-1 induction, cleavage of microtubule-associated protein 1 light chain 3 and autophagolysosome formation, and apoptosis, respectively [93].

## 3. Role of Antioxidant Therapy in Myocardial Infarction

**Figure 2:**
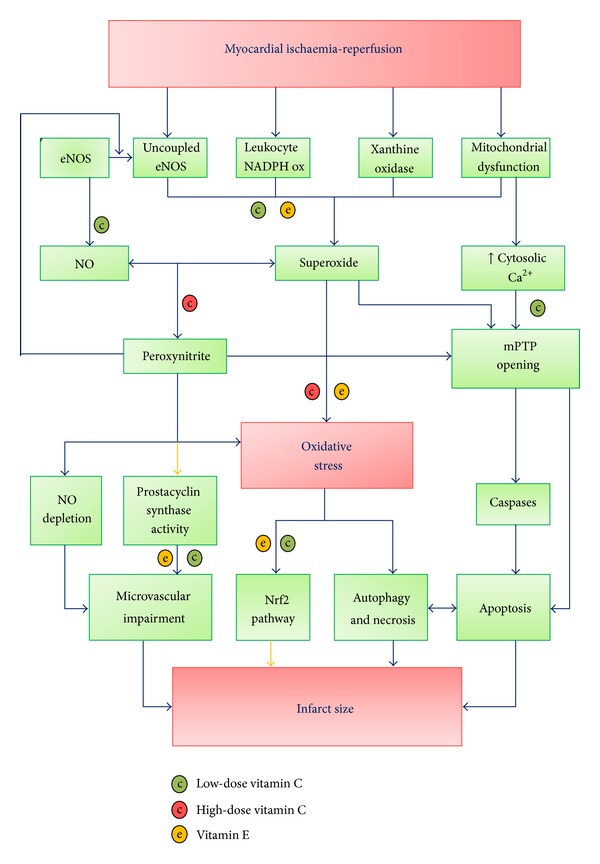
Hypothesis accounting for the acute myocardial infarct size occurring in ischemia-reperfusion through molecular models based on the role of oxidative stress. Abrogation of the deleterious processes by vitamins C and E. Arrow color code: blue stands for “promotion”; yellow for “inhibition”: mPTP mitochondrial permeability transition pore; NO; nitric oxide; eNOS; endothelial nitric oxide synthase; NADPH, reduced nicotinamide adenine dinucleotide phosphate; NADPH ox, oxidized nicotinamide adenine dinucleotide phosphate; Nrf2, nuclear factor (erythroid-derived 2)-like 2. Adapted from [15], with permissions.

The therapeutic effects of vitamins C and E will be discussed in the following section. A summary of these effects can be found in Figure 2.

### 3.1. Vitamin E

The potential therapeutic effects of vitamin E in AMI can be comprised of biological actions such as antioxidant and anti-inflammatory effects, as well as a synergism with other antioxidant molecules. Indeed, vitamin E, mainly *α*-tocopherol, is the major peroxyl radical scavenger in biological lipid-phases such as membranes or LDL [94, 95]. The antioxidant action has been ascribed to its ability to act chemically as a lipid based free radical chain-breaking molecule, thereby inhibiting lipid peroxidation through its own conversion into an oxidized product, *α*-tocopheroxyl radical. *α*-Tocopherol can be restored by reduction of the *α*-tocopheroxyl radical with redox-active reagents like vitamin C or ubiquinol [96]. Otherwise, the tocopheroxyl radical can react with lipids to generate lipid radicals. Therefore, therapeutic uses of *α*-tocopherol probably require coantioxidants such as vitamin C to have a beneficial effect [97]. The antioxidant effect of vitamin E is not limited to a role of lipid phase ROS scavenger, as it can increase glutathione peroxidase activity [98] and diminish ROS production via downregulating NADPH oxidase [99]. This antioxidant vitamin also has anti-inflammatory effects, by inhibiting the transcriptional activity of NF-*κ*B, a factor able to trigger the expression of proinflammatory genes [100]. The inhibition of the transcriptional activity of NF-*κ*B should also contribute as an anti-inflammatory effect [101]. In addition, *α*-tocopherol has been shown to inhibit many key events in inflammation such as, but not limited to, platelet aggregation [97] and the release of pro-inflammatory cytokines [102].

It is of interest to mention that vitamin E can exert positive effects reported in clinical studies of revascularization surgeries, such as those of the lower extremities [103], kidney transplantation [104], liver surgery [105], and aortic aneurysm repair [106]. Furthermore, preoperative administration of vitamin E is safe, and this treatment may have beneficial effects by reducing the impact of ischemia-reperfusion injury in liver surgery [107]. Although homologous studies in AMI are still lacking, an amelioration of microvascular impairment in myocardial tissue should be expected, as an effect of pretreatment with vitamin E. However, this effect needs to be tested by further studies.

### 3.2. Vitamin-C Scavenging Is Concentration Dependent and Requires Intravenous Administration

Plasma vitamin C concentrations do not exceed 100 *μ*mol/L with the ingested amounts found in foods. Even with supplementation approaching maximally tolerated doses, ascorbate plasma concentrations are always <250 *μ*mol/L and frequently <150 *μ*mol/L. By contrast, intravenously injected ascorbate can lead to concentrations of 25–30 mmol/L that are safely achieved [108]. Therefore, in settings accompanied by oxidative stress, such as the myocardial ischemia-reperfusion events, a major beneficial effect of oral administration of vitamin C in the prevention of oxidative damage should not be expected, and intravenous infusion could be considered with this purpose. Indeed, superoxide reacts with NO at a rate 10^5^-fold greater than the rate at which superoxide reacts with ascorbic acid [8]. As a consequence, to displace the binding of superoxide anion and NO 10 mmoles/L ascorbate would be needed. 

Likely, intracellular levels of ascorbate may not be reflected accurately in its plasma levels since, due to ascorbate transporters, it is accumulated in cells against a concentration gradient by all tissues other than red blood cells [109]. In some tissues, ascorbate levels may exceed the plasma concentrations by as much as 100-fold [110].

### 3.3. Relationship between Plasmatic Doses and Intracellular Myocardial Concentration: Role of Transporters

Vitamin C is present in the organism in two biological important forms: the reduced form, ascorbic acid, and the oxidized form, dehydroascorbic acid (DHA). Both chemical forms are transported intracellularly [110], a process that requires the participation of specific transporters at the level of the plasma membrane [110]. Once inside the cells, DHA is rapidly reduced to ascorbic acid [111, 112].

Two Na^+^-dependent vitamin C transporters (SVCT1 and SVCT2) serve the function of entering ascorbic acid into the cell [113] while DHA transporters are members of the GLUT family of facilitative glucose transporters, with GLUT1, GLUT3, and GLUT4 being DHA transporters [110]. Human myocardium contains GLUT1, GLUT3, and GLUT4 [114].

SVCT1 is confined to epithelial systems including intestine, kidney, and liver, whereas SVCT2 has a widespread location in the body and serves as a host of metabolically active and specialized cells and tissues [113], including the heart [115, 116]. It has been proposed that among the roles of SVCT2 is to provide l-ascorbic acid to protect metabolically active cells from oxidative stress [113].

In accordance with the previously mentioned studies, Guaiquil et al. report an *in vitro* study where adult rat cardiomyocytes incubated for 30 min with 5 mM DHA accumulated 39 mM ascorbic acid [117]. 

### 3.4. Vitamin C and Synergistic Effects with Vitamin E

Vitamin C, ascorbic acid or ascorbate, is a reducing agent that serves as a one-electron donor, generating semidehydroascorbate. When it acts as an antioxidant or enzyme cofactor, it becomes oxidized to DHA [118]. Ascorbate counteracts and prevents the oxidation of lipids, proteins, and DNA, subsequently protecting their structure and biological function. Together with glutathione, ascorbic acid constitutes a primary line of defense against ROS [119]. 

Ascorbate in aqueous compartments can recycle *α*-tocopherol in membranes by reducing the *α*-tocopheroxyl radical back to *α*-tocopherol [120]. Accordingly, ascorbate has been shown to recycle *α*-tocopherol in lipid bilayers [121] and erythrocytes [122].

The antioxidant effect of ascorbic acid is not limited to its ability to scavenge ROS. Ascorbate can diminish ROS production through downregulation of NADPH oxidase. The therapeutic potential of vitamin C becomes clear if it considered that the major source of ROS in AMI is their enzymatic production via NADPH oxidase [32, 45, 46]. Vitamin C also suppresses NF-*κ*B activation [123]. 

In addition, vitamin C prevents the oxidation of tetrahydrobiopterin, a cofactor of NO synthase that is highly sensitive to oxidation. When tetrahydrobiopterin is oxidized, eNOS activity becomes uncoupled, resulting in the production of superoxide instead of NO, thus enhancing the oxidative damage [124].

As it was previously mentioned, concentrations acquired through oral administration of vitamin C doses are not enough to scavenge superoxide anion. Therefore, i.v. administration is required for this purpose.

Impaired microcirculatory reperfusion is improved by vitamin C infusion in hypertension [125] diabetes mellitus [126] and in patients undergoing elective PCA, suggesting that oxidative stress is implicated in such a phenomenon [127]. Also, in patients subjected to thrombolysis following AMI, SOD in the blood was found to be significantly reduced, whereas the activity of the oxidant enzyme, xanthine oxidase, and malondialdehyde levels were found to be significantly increased. However, oral supplementation of vitamin C to the postreperfusion patients restored these parameters back to normal or near normal levels [128]. 

Even though vitamins C and E exert their individual biochemical effects in water or lipid phases, respectively, they also can interact with each other at the level of interphases, giving rise to synergistic effects of restoring *α*-tocopherol from *α*-tocopheroxyl radical [13]. In vitamin-E-supplemented rat hearts, *α*-tocopherol diminishes rapidly without the addition of vitamin C during reperfusion [129].

## 4. Clinical Experiences of Antioxidant Treatment of Myocardial Infarction

Experimental studies based on the pathogenic role of ROS and reactive nitrogen species (RNS) in myocardial damage following ischemia-reperfusion events have given promising results for antioxidant cardioprotection. Therefore, it should be expected that treatments with exogenous antioxidant agents could protect the heart against lethal reperfusion injury in clinical models. However, although a number of strategies have been devised to ameliorate this injury, the beneficial effects in the clinical settings have been disappointing up to date [15]. Thus, clinical trials designed to study cardioprotection by long-term administration of vitamins C and E have failed to demonstrate beneficial effects [130–137]. Either thrombolytic therapy or primary percutaneous coronary intervention has proved to be the most effective therapeutic intervention for reducing acute myocardial ischemic injury. However, reperfusion itself can induce cardiomyocyte impairment of structure and function. Consequently, myocardial stunning and even cell death will occur, what is known as myocardial reperfusion injury, for which there is still no effective therapy. Moreover, studies in animal models of AMI suggest that lethal reperfusion accounts for up to 50% of the final myocardial infarct size [7], a damage likely to be preventable. 

Randomized, double-blind, placebo-controlled trials with antioxidant therapy using L-carnitine [23] and Coenzyme Q10 [22] as an oral treatment after AMI have suggested a reduction in infarct size and improvement of the clinical outcomes in treated patients (Table 1). Nevertheless these therapies could not primariy prevent lethal reperfusion injury, because of the slow enteric absorption of L-carnitine [138] and Coenzyme Q10 [139]. Some studies have suggested that antioxidant agents attenuate left ventricular remodeling following AMI. Accordingly, in patients with AMI who had undergone primary percutaneous transluminal coronary angioplasty, pretreatment with allopurinol, a xanthine oxidase inhibitor, resulted in effective inhibition of ROS generation and significant improvement of left ventricular ejection fraction at 6 months after PTCA [27]. More recently, administering the ROS scavenger edaravone to patients with AMI immediately prior to reperfusion significantly reduced infarct size and reperfusion arrhythmias [25]. In this study the free radical scavenger was given intravenously and prior to the onset on the reperfusion therapy, thus accounting for the role of oxidative stress in lethal reperfusion injury. However, other attempts, such as intravenous bolus of superoxide dismutase [26], showed no beneficial effect on patients outcome. The authors report that it is possible that a clinically significant benefit might have been missed with such a small sample size because of the heterogeneity of intercoronary collaterals, vascular risk regions, and other important uncontrolled variables. In brief, antioxidants, as well as numerous cardioprotective strategies for reducing lethal reperfusion injury, have failed to provide any benefit to patients during reperfusion heart damage [7]. 

In regard to the therapeutic use of vitamins C and E with purposes of cardioprotection, although the scientific rationale, epidemiologic data, and retrospective studies have been persuasive, prospective, randomized, placebo-controlled trials have not verified their actual benefit in human diseases [140]. Only one randomized, double-blind, placebo-controlled study has been published using vitamins C and E before the reperfusion therapy [17]. They used 1000 mg/12 hr infusion of vitamin C followed by 1200 mg/24 hr orally and vitamin E (600 mg/24 hr) for 30 days. Results suggest that supplementation with these antioxidants seems to positively influence the clinical outcome of patients with AMI, in terms of composite of in-hospital cardiac mortality, nonfatal new myocardial infarction, ventricular tachycardia, ventricular fibrillation, asystole or shock, and pulmonary edema. Furthermore in a retrospective analysis of the aforementioned data [18] a significant reduction in 30-day cardiac mortality in diabetic patients treated with vitamins C and E has been found. In patients without diabetes, the administration of vitamins had no such effect on cardiac mortality. This result on diabetic patients with AMI seems to be particularly reasonable because of the increased ROS formation known to occur in these patients [141]. It should be noted that the authors acknowledged that the dose of vitamin C used only raised plasma levels to 0.1 mmol/L. Thus, the most important function to abrogate oxidative stress-dependent processes cannot be achieved by vitamin C doses used in this study. Indeed, it is necessary to reach plasma levels of ascorbate about 10 mmol/L to prevent chemical reaction of NO and superoxide anion, otherwise resulting in a highly peroxidant pathway [8]. 

It is of interest to remark that up to date the available clinical trials have been designed with significant methodological deficiencies that demand cautious interpretation of these results. Nevertheless, some beneficial effects derived from the biological properties of antioxidant vitamins could be expected in the patients subjected to these protocols, depending on the dose and administration manner. Taking into consideration the high reactivity of ROS, their short life span, their continuous production in close proximity to biological targets, and their ability to be modified into other more reactive species, one realizes that, in order to cope with these deleterious metabolites, the antioxidant therapy should be administered to the body continuously, in high concentrations, and targeted to the biological site susceptible to oxidative damage. In addition, to scavenge ROS efficiently, antioxidants must be present at the location of radical formation in order to compete with the biological target [51]. Therefore, antioxidant therapy should be designed carefully [142–145]. On the other hand, an understanding of the mechanism of the activity of scavengers, including their mutual collaboration, synergistic activity, and interrelationships, prompts the suggestion that the antioxidant be given in combinations, such as preparations of multiscavenger in both oxidized and reduced forms and with no transition metals in the formulations. They should be designed in appropriate pharmaceutical dosage forms such as sustained-release formulations. One has to be aware of their potential side effects and their upper toxic dose, which can easily be reached, because these compounds are widely distributed in our diet [51].

## 5. Conclusions and Perspectives

Cumulated data strongly suggest the major contribution of ROS to the development of oxidative damage in myocardial infarction followed by revascularization, a clinical model of oxidative stress. Nevertheless, the clinical trials aimed to test the therapeutic role of antioxidants in reperfusion damage have failed to find a beneficial effect. These disappointing results for the case of vitamins C and E could be explained on the basis of the methodological design of the protocols. Oral doses of vitamin C are not suitable to reach plasma levels high enough to scavenge ROS (i.e., above 10 mmoles/L). Although these levels could be achievable by massive infusion of ascorbate, up to date no protocols have been performed with this purpose. It is of particular relevance to consider that (i) vitamin E, mainly *α*-tocopherol, is the major peroxyl radical scavenger in biological lipid phases such as membranes or LDL; (ii) *α*-tocopherol can be restored by reduction of the *α*-tocopheroxyl radical with redox-active reagents like vitamin C; (iii) infusion of ascorbate at a rate high enough to scavenge ROS could offer an unexplored therapeutic opportunity in short-term surgical procedures involving major risk of oxidative stress and its consequences; (iv) although a scavenging effect at oral doses of vitamin C should not be expected, decreased ROS production could result from its ability to downregulate NADPH oxidase activity, an effect shared by vitamin E; (v) stabilization of tetrahydrobiopterin, a cofactor of eNOS, could also be achieved by oral doses of vitamin C; otherwise the enzyme could produce superoxide instead of NO; and (vi) the whole molecular effects of vitamins C and E could account for an abrogation of the microvascular adverse events occurring in the percutaneous coronary angioplasty, as well as in other surgical procedures involving ischemia-reperfusion events. It is noticeable that these safe, easily available, low cost naturally occurring substances could improve the clinical outcome of patients subjected to percutaneous angioplasty, a novel view likely to give rise to the performance of clinical trials devised to demonstrate the validity of this paradigm.

## Figures and Tables

**Table 1:** Cardioprotective strategies using antioxidant vitamins C and E and other antioxidants in acute myocardial infarction.

Details of Study	Study	*n*	Results	References
Vitamins C and E				
Vitamin C (1000 mg/12 h infusion) followed by 1200 mg/24 h orally and vitamin E (600 mg/24 h).	Randomized, double-blind, placebo-controlled, multicenter trial.	800	Improvement in mortality and clinical outcomes.	Jaxa-Chamiec et al. (MIVIT trial) [17]
A retrospective analysis of the influence of vitamins C and E on 30-day cardiac mortality in patients with or without DM.	Retrospective study from MIVIT trial.	800 [122 (15%) DM]	Reduction in cardiac mortality in DM patients treated. No significant differences in nondiabetic patients.	Jaxa-Chamiec et al. [18]
Vitamins C and E (600 mg/24 h each) orally on the first day of AMI and lasting for 14 days.	Randomized, double-blind, placebo-controlled trial.	37	Baseline QTd was similar in both groups. Significant decrease in exercise-induced QTd in treated group.	Bednarz et al. [19]
Vitamin A (50,000 IU/24 h), vitamin C (1,000 mg/24 h), vitamin E (400 mg/24 h), and beta-carotene (25 mg/24 h)	Randomized, double-blind, placebo-controlled trial.	125	Reduction in mean infarct size assessed by cardiac enzymes. Improved clinical outcomes.	Singh et al. (the Indian experiment of infarct survival-3) [20]
Vitamins C and E, each 600 mg/24 h orally for 14 days.	Randomized trial.	61	Less ECG alterations in treated patients.	Chamiec et al. [21]

Other Antioxidants				
Oral treatment with coenzyme Q10 (120 mg/24 h) for 28 days, administered within 3 days of the onset of symptoms.	Randomized, double-blind, placebo-controlled trial.	144	Angina pectoris, total arrhythmias, and poor left ventricular function were significantly reduced.	Singh et al. [22]
Oral L-carnitine (2 g/24 h) for 28 days.	Randomized, double-blind, placebo-controlled trial.	101	Significant reduction in mean infarct size assessed by cardiac enzymes.	Singh et al. [23]
High-dose N-acetylcysteine (2 × 1,200 mg/24 h) for 48 h, plus optimal hydration.	Randomized, single-blind, placebo-controlled trial.	251	No differences in any of the end point with N-acetylcysteine or placebo.	Thiele et al. [24]
30 mg edaravone intravenously before reperfusion.	Randomized, placebo-controlled trial.	101	Significant reduction in reperfusion arrhythmia and mean infarct size assessed by cardiac enzymes.	Tsujita et al. [25]
Intravenous bolus of superoxide-dismutase (10 mg/kg of body weight) followed by a 60 min infusion of 0.2 mg/kg/min before PCI.	Randomized, placebo-controlled trial.	120	No significant differences.	Flaherty et al. [26]
Allopurinol (400 mg) administered orally just after the admission (approximately 60 min before reperfusion).	Randomized trial.	38	Slow flow in the recanalized coronary artery after PTCA occurred less frequently.	Guan et al. [27]

*AMI: acute myocardial infarction; DM: diabetes mellitus; PCI: percutaneous coronary intervention; QTd: QT dispersion in electrocardiogram; ECG: electrocardiogram; PTCA: percutaneous transluminal coronary angioplasty.

## Abbreviations

AMI: Acute myocardial infarction
AMPK: 5′Adenosine monophosphate-activated protein kinase
CAT: Catalase
CRP: C-reactive protein
DHA: Dehydroascorbic acid
GSH: Reduced glutathione
GSH-Px: Glutathione peroxidase
H_2_O_2_: Hydrogen peroxide
IL-6: Interleukin-6
IL-1*β*: Interleukin-1*β*
MAPK: Mitogen-activated protein kinase
MCP-1: Chemokine monocyte chemoattractant protein-1
MCPIP: MCP-1-induced protein
mPTP: Mitochondrial permeability transition pore
MPO: Myeloperoxidase
mTOR: Mammalian target of rapamycin
NF-*κ*B: Nuclear factor *κ*-light-chain-enhancer of activated B cells
NO: Nitric oxide
OH*·*: Hydroxyl radical
ONOO^−^: Peroxynitrite
PCA: Percutaneous coronary angioplasty
RNS: Reactive nitrogen species
ROS: Reactive oxygen species
SOD: Superoxide dismutase
TNF-*α*: Tumour necrosis factor-*α*.
